# Assessment of Molecular Measures in Non-FXTAS Male Premutation Carriers

**DOI:** 10.3389/fgene.2018.00302

**Published:** 2018-08-22

**Authors:** Reem R. Al Olaby, Hiu-Tung Tang, Blythe Durbin-Johnson, Andrea Schneider, David Hessl, Susan M. Rivera, Flora Tassone

**Affiliations:** ^1^Department of Biochemistry and Molecular Medicine, UC Davis Medical Center, University of California, Davis, Davis, CA, United States; ^2^Department of Biostatistics, University of California, Davis, Davis, CA, United States; ^3^Department of Pediatrics, UC Davis Medical Center, University of California, Davis, Davis, CA, United States; ^4^MIND Institute, UC Davis Medical Center, Sacramento, CA, United States; ^5^Department of Psychiatry and Behavioral Sciences, UC Davis Medical Center, University of California, Davis, Davis, CA, United States; ^6^Neurocognitive Development Lab, Department of Psychology, UC Davis Center for Mind and Brain, University of California, Davis, Davis, CA, United States

**Keywords:** *FMR1*, ASFMR1, transcription, premutation, FXTAS, CATSYS, splicing isoforms

## Abstract

Approximately 30–40% of male and 8–16% of female carriers of the Fragile X premutation will develop a neurodegenerative movement disorder characterized by intentional tremor, gait ataxia, autonomic dysfunction, cognitive decline, and Parkinsonism during their lifetime. At the molecular level, premutation carriers have increased expression levels of the *FMR1* and the antisense *FMR1* (*ASFMR1)* mRNAs. Both genes undergo alternative splicing giving rise to a number of different transcripts. Alteration in the alternative splicing process might be associated with FXTAS. In this study, we have investigated the correlation between objective measures of movement (balance and tremor using the CATSYS battery) and the expression of both the *FMR1* and the *ASFMR1* genes. In addition, we investigated whether their expression level and that of the *ASFMR1* 131 bp splice isoform could distinguish between premutation carriers with FXTAS and non-FXTAS premutation carriers. Confirming previous findings, the expression levels of transcripts at the *FMR1* locus positively correlated with the CGG repeat number and significantly differentiated the premutation carriers from the control groups. Furthermore, premutation carriers with and without FXTAS, showed a significant difference in the expression level of the *ASFMR1* 131 bp splice isoform when compared to age and gender matched controls. However, there was no significant difference in the *ASFMR1* 131 bp splice isoform expression level when comparing premutation carriers with and without FXTAS. Finally, our results indicate significant group differences in CATSYS dominant hand reaction time and postural sway with eyes closed in premutation carriers without FXTAS compared to controls. In addition, a significant inverse association between the tremor intensity and the expression level of *ASFMR1* 131 bp splice isoform in premutation carriers compared to controls, was observed, suggesting a potential role in the pathogenesis of FXTAS.

## Introduction

Individual carriers of a premutation allele in the *FMR1* gene (55–200 CGG repeats) are at risk of developing Fragile X-associated tremor/ataxia syndrome (FXTAS) a late onset neurodegenerative disorder characterized by intentional tremor, gait ataxia, autonomic dysfunction, and Parkinsonism (Tassone and Hall, 2016). Cognitive decline, particularly frontal executive dysfunction, is also very common (Allen et al., 2008; Grigsby et al., 2008). Approximately 46% of males and 17% of females will develop and be affected by FXTAS during their lifetime. The onset of the motor signs in men is typically in the early 60s (Tassone et al., 2007) and the penetrance is age-related, such that 75% of men ≥80 years of age are affected (Jacquemont et al., 2004). Patients with FXTAS have several types of motor dysfunction, hence they are considered within the combinational movement disorders (Hall et al., 2014).

A higher frequency of a number of phenotypes including psychiatric conditions, dysautonomia, sleep apnea, hypertension, migraine, rheumatologic conditions, endocrine diseases, seizures, peripheral neuropathy, immune mediated conditions (such as fibromyalgia and hypothyroidism) has also been described in premutation carriers (reviewed in Hagerman and Hagerman, 2016).

Several molecular mechanisms have been proposed to contribute to the different phenotypes observed in the *FMR1*- associated disorders. One of the main mechanisms proposed to lead to the premutation pathogenesis is RNA toxicity due to the 2 to 8-fold increase in the *FMR1* mRNA expression levels observed in premutation carriers (Tassone et al., 2000; Kenneson et al., 2001; Allen et al., 2005). RNA toxicity leads to protein sequestration where the expanded CGG repeat sequesters a number of CGG binding proteins, hence, partially or fully impairing their normal function in the cell (Hagerman and Hagerman, 2015). Recently, a non-AUG initiated (RAN) translation, where a polyglycine-containing protein, FMRpolyG is generated by initiating at non-AUG codons located upstream of the CGG-repeat region (Todd et al., 2013), has been proposed as a key mechanism contributing to neurodegeneration. Further, DNA damage has been proposed as a pathological model due to the formation of co-transcriptional R-loops which trigger a DNA damage response (DDR) leadings to neuronal death (Ginno et al., 2013; Hamperl and Cimprich, 2014; Loomis et al., 2014; Julie and Karlene, 2015).

In an effort to elucidate other mechanistic pathways that might be involved in FXTAS, Ladd et al. (2007) identified a novel gene, the antisense *FMR1* (*ASFMR1*), the main identified non-coding RNA (ncRNA) at the *FMR1* locus. The gene includes the CGG repeat region of the *FMR1* gene in the antisense orientation. Its expression is driven by two promoters that are flanked by CTCF-binding sites, the *FMR1* bidirectional promoter and a second one located in the second intron of the *FMR1* gene (Ladd et al., 2007). Similarly, to the *FMR1* gene, the levels of expression of *ASFMR1* mRNA are elevated in the cells derived from premutation carriers compared to controls (Ladd et al., 2007; Loesch et al., 2011) while no expression is detected in patients with Fragile X syndrome. Furthermore, the *ASFMR1* transcript undergoes to premutation-specific alternative splicing which might be potentially associated with FXTAS and other *FMR1* associated disorders (Ladd et al., 2007; Hall et al., 2017).

Loesch et al. (2011) showed that the elevated sense/antisense *FMR1* transcript levels in the gray zone (40–54 CGG repeats) and in premutation carriers might contribute to the development of a parkinsonian phenotype due to mitochondrial dysfunction that leads to progressive neurodegeneration. A recent study by Hall et al. (2017) showed that both male and female premutation carriers had higher expression levels of *ASFMR1* splice isoform corresponding to the isoform described by Ladd et al. (2007), compared to controls after adjusting for age, confirming previous finding Ladd et al. (2007). Although the authors suggested *ASFMR1* splice isoform as a predictor of FXTAS, they reported that there was no significant difference in the expression levels between non-FXTAS premutation carriers and FXTAS patients (Hall et al., 2017).

Besides the molecular measures, clinical measures have a significant role in monitoring premutation carriers with and without FXTAS. A computerized coordination-tremor-balance test system (CATSYS) is one of the important clinical assessments for FXTAS. It is a quantitative neurological test battery used to quantify movement abnormalities (tremor and ataxia) recording five main neuromotor control measures. Previous studies reported on the sensitivity of the test in identifying preclinical symptoms of FXTAS (Allen et al., 2008) and on its ability to differentiate between premutation carriers with FXTAS and controls (Aguilar et al., 2008; Narcisa et al., 2011).

In this study, we investigated the correlation between levels of mRNA expression of the *FMR1* locus (mRNA expression levels of the *FMR1* and of the *ASFMR1* genes and of the *ASFMR1* 131 bp splice isoform) and a selected group of measures in the CATSYS associated with the core symptoms of FXTAS (tremor and balance) in the non-FXTAS premutation carriers compared to controls. In addition, we investigated whether there was a differential transcript profile at the *FMR1* locus expression between premutation carriers with and without FXTAS compared to controls. The main aim of the study was to determine whether a combination of neurological and molecular measures could help predict the prognosis of non-FXTAS male premutation carriers.

## Materials and Methods

### Participants

Individuals were recruited through the MIND Institute Fragile X Research and Treatment Center, by postings in the community, from flyers posted through the National Fragile X Foundation, from over 1,200 extended pedigrees of probands with Fragile X-associated disorders seen for clinical care or for being participants in research studies involving non-FXTAS premutation carriers, carried out at the MIND Institute. Participants provided informed consent according to protocols approved by the UC Davis Institutional Review Board. Biological samples were collected under protocols approved by the UC Davis Institutional Review Board.

Participants included 52 non-FXTAS male premutation carriers (mean age = 59.3, *SD* = 8.3) and 26 healthy controls (mean age = 55.6, *SD* = 8.7) (**Table 1**).

**Table 1:** Patient demographics by group.

	Control (*n* = 26)	Premutation (*n* = 52)	*p*-value	Adjusted *p*-value
Age (years)			0.1488	0.4408
*N*	26	46		
Mean (*SD*)	55.6 (8.7)	59.3 (8.3)		
Median (range)	55.5 (40-68)	61 (40-78)		
Primary race (n, %)			0.4408	0.4408
Asian	1 (3.8%)	1 (1.9%)		
Black or African American	1 (3.8%)	0		
White	22 (84.6%)	42 (80.8%)		
Not reported	2 (7.7%)	9 (17.3%)		
Primary ethnicity			0.3588	0.4408
Hispanic or Latino	3 (11.5%)	2 (3.8%)		
Not hispanic or latino	18 (69.2%)	32 (61.5%)		
Not reported	5 (19.2%)	18 (34.6%)		
Handedness			0.2442	0.4408
Right	22 (84.6%)	43 (82.7%)		
Left	4 (15.4%)	3 (5.8%)		
Not reported	0	6 (11.5%)		
Education level			0.3798	0.4408
High school/GED	2 (7.7%)	2 (3.8%)		
Partial college	3 (11.5%)	11 (21.2%)		
BA/BS	11 (42.3%)	12 (23.1%)		
MA/MS/PhD/MD	10 (38.5%)	20 (38.5%)		
Not reported	0	7 (13.5%)		
Yearly household income			0.2827	0.4408
25–50 K	2 (7.7%)	2 (3.8%)		
50–75 K	2 (7.7%)	7 (13.5%)		
75–100 K	5 (19.2%)	4 (7.7%)		
100–150 K	8 (30.8%)	12 (23.1%)		
150–250 K	5 (19.2%)	6 (11.5%)		
>250 K	1 (3.8%)	9 (17.3%)		
Not reported	3 (11.5%)	12 (23.1%)		
Marital status			0.0728	0.4408
Married/partner	19 (73.1%)	42 (80.8%)		
Divorced	2 (7.7%)	2 (3.8%)		
Single	4 (15.4%)	1 (1.9%)		
Not reported	1 (3.8%)	7 (13.5%)		

In addition, we investigated the difference in expression levels of transcripts at the *FMR1* locus in 15 trios. Each trio was age-matched, and included a male premutation carrier with FXTAS (FXTAS stage 4 or 5) (*n* = 15), a non-FXTAS male premutation carrier (FXTAS stage 0 or 1) (*n* = 15) and a healthy control (*n* = 15) (total of 45 participants).

Group status for each participant was confirmed through DNA testing as having 55–200 CGG repeats (carriers of the *FMR1* premutation), or having 5–44 CGG repeats (normal range, comparison group). Individuals who had a gray zone allele (45–54 CGG repeats) or a full mutation (<200 CGG repeats) were not included in this study.

### Clinical Measures

CATSYS included various tasks grouped into five existing categories (Després et al., 2000): postural tremor, intentional tremor, postural sway, manual coordination, and reaction time. All tasks were appended to the standard CATSYS protocol and analyzed accordingly.

The CATSYS measures were administered as described in Aguilar et al. (2008). Postural tremor was measured per the CATSYS protocol. For both dominant and non-dominant hands, the patient was asked to grasp a Tremor Pen^®^ (it contains a biaxial micro-accelerometer to measure the movement in plane perpendicular to the axis of the pen) and hold it as steadily as possible four inches in front of the navel. For the intention tremor performance task, the patient was asked to grip the pen in the same way as in the postural tremor task, yet not in a steady position, but to alternately tap the centers of two points located on either side of the computer screen, designated as points A and B, for each hand.

As for the postural sway protocol, patients were asked to stand on the force plate for 30 and 10 s, once with the eyes open and once closed. Manual hand coordination was measured by asking patients to rhythmically tap the drum in time with sounds generated by the CATSYS program. The testing using this category was completed with assessing the finger coordination, which included the rhythmic tapping of the right and left index finger. Finally, the reaction time for response, which was an auditory stimulus, in this case was recorded using the reaction handle of the CATSYS system. The duration for testing each hand was 40 s where the auditory stimuli were triggered at random intervals.

### Molecular Measures

#### DNA and RNA Isolation

Genomic DNA was isolated from peripheral blood lymphocytes (5 ml of whole blood using standard methods; Qiagen, Valencia, CA, United States). The CGG size of the premutation and normal alleles were obtained using a combination of Southern Blot and PCR analysis. For Southern blot analysis, 5–10 μg of isolated genomic DNA was digested with EcoRI and NruI. Probe hybridization used the FMR1-specific dig-labeled StB12.3. Details are as previously described (Tassone et al., 2008). PCR analysis was performed using the AmplideX PCR/CE *FMR1* Reagents (Asuragen, Inc.) as described in Filipovic-Sadic et al. (2010). Total RNA was isolated from 2.5 ml of peripheral blood collected in PAXgene Blood RNA tubes using the PAXgene Blood RNA Kit (Qiagen, Valencia, CA, United States) and quantified using NanoDrop. cDNA synthesis reaction was as described by Tassone et al. (2000).

#### Measures of mRNA Expression Levels by qRT-PCR

qRT-PCR was performed using both Assays-on-Demand from Applied Biosystem (Applied Biosystems, Foster City, CA, United States) and custom designed TaqMan primers and probe assays to measure transcripts expression levels. Custom designed primers and probe were used for normalization (Tassone et al., 2000). Custom designed primers and probe were also designed to quantify the *ASFMR1* gene and the *ASFMR1* splice isoform (Ladd et al., 2007).

### Statistical Analysis

Left- and right-handed clinical measures were converted to dominant and non-dominant based on the patient’s handedness; if handedness information was missing measures from the right hand were used for the dominant side. A patient with a CGG repeat ranging from 110 to 130 was assigned a repeat length of 120 for analysis purposes.

By visual inspection, CATSYS data deviated substantially from a normal distribution both on the original scale and on the log scale (other transformations were not considered, as model coefficients estimated based on these are rather less interpretable than the aforementioned scales). Therefore, non-parametric methods were used throughout for analysis of these data.

Age, clinical measures, and molecular measures were compared between groups using Wilcoxon rank sum tests. Distributions of categorical demographic characteristics were compared between groups using Fisher’s exact test. Correlations were estimated and tested using the Spearman (non-parametric) correlation. Clinical measures were compared between visits using the Wilcoxon signed-rank test.

In the trios study, expression levels of *ASFMR1*, the splice isoform, and *FMR1* were compared among premutation participants with FXTAS, premutation participants with non-FXTAS, and control subjects using linear models. Age and CGG repeat number were adjusted for by including these as covariates in each model. *Post hoc* pairwise comparisons between groups were conducted using the Tukey HSD method. Analyses were conducted using R, version 3.4.2 (R Core Team, 2017).

*p*-values were adjusted for multiple testing within each table using the Benjamini–Hochberg method.

## Results

### Demographics

In this study, among the total 78 subjects, 82.1% (*n* = 64) were Caucasian and 38.5% (*n* = 30) had a higher degree education (MA/MS/PhD/MD). In addition, 83.3% (*n* = 65) were right hand dominant. The demographics data showed no statistical significant difference between any of the groups (**Table 1**).

As for the trio study, participants were individually matched for CGG repeats and age; where the mean age (SD) for the 15 controls was 63 (±8.9), for the 15 FXTAS premutation carriers was 65 (±8), and for the 15 non-FXTAS premutation carriers was 62 (±8). The mean (SD) for the CGG repeat number was 98.47 (±15.8) in male premutation carriers with FXTAS, 90.27 (±22.8) in non-FXTAS premutation carriers and 29.67 (±4.4) in controls (**Table 2**). The mean (SD) of the *FMR1* mRNA was 2.9 (±0.6) in the premutation with FXTAS, 1.9 (±1.1) in the non-FXTAS premutation and 1.4 (±0.4) in controls. The mean of the *ASFMR1* mRNA was 0.51 (±0.3) in the premutation with FXTAS, 0.53 (±0.3) in the non-FXTAS premutation and 0.3 (±0.1) in controls. The mean of the *ASFMR1* 131 bp splice isoform mRNA was 1.9 (±1.6) in the premutation with FXTAS, 1.7 (±2.1) in the non-FXTAS premutation and 0.1 (±0.1) in controls.

**Table 2:** Trio study subjects.

Trio #	Category	CGG #	Age	FXTAS stage
1	FXTAS	88	64	5
	Asymp Pre	75	66	1
	Control	31	65	0
2	FXTAS	104	56	4
	Asymp Pre	98	59	1
	Control	21	50	0
3	FXTAS	119	67	5
	Asymp Pre	105	64	0
	Control	28	66	0
4	FXTAS	102	69	4
	Asymp Pre	110	57	1
	Control	29	65	0
5	FXTAS	133	54	4
	Asymp Pre	141	52	1
	Control	28	53	0
6	FXTAS	96	61	4
	Asymp Pre	95	60	1
	Control	33	61	0
7	FXTAS	80	79	4
	Asymp Pre	80	71	0
	Control	32	76	0
8	FXTAS	82	57	4
	Asymp Pre	84	51	0
	Control	32	52	0
9	FXTAS	103	58	4
	Asymp Pre	128	53	0
	Control	29	55	0
10	FXTAS	100	56	4
	Asymp Pre	80	56	0
	Control	28	55	0
11	FXTAS	88	66	4
	Asymp Pre	85	63	1
	Control	27	64	0
12	FXTAS	83	70	4
	Asymp Pre	77	69	1
	Control	42	70	0
13	FXTAS	121	76	4
	Asymp Pre	67	74	1
	Control	28	73	0
14	FXTAS	83	75	4
	Asymp Pre	59	76	0
	Control	30	79	0
15	FXTAS	95	66	4
	Asymp Pre	70	64	1
	Control	27	64	0

### A Subset of CATSYS Clinical Measures Differentiate Between Non-FXTAS Premutation Carriers and Controls

Three CATSYS performance measures were found to be significantly different between non-FXTAS premutation carriers and controls; the reaction time test for dominant hand (Average reaction time) (*p* = 0.0094), the 30 s postural sway test with eyes open (*p* = 0.0176), and the 10 s postural sway test with eyes closed (*p* = 0.0094) (**Table 3**). This means that both the reaction time and the sway area are significantly increased in non-FXTAS premutation carriers compared to controls.

**Table 3:** CATSYS clinical measures in both normal and premutation carrier groups.

	Control		Premutation		*p*-value	Adjusted *p*-value
	Mean (*SD*)	Median (range)	Mean (*SD*)	Median (range)		
Reaction time test for dominant hand: average reaction time	0.204 (0.02)	0.2 (0.159-0.239)	0.23 (0.032)	0.23 (0.167-0.295)	**0.0011**	**0.0094**
Sway closed 30: sway area	454 (402)	403 (5-1703)	802 (580)	653 (11-2,300)	**0.0033**	**0.0176**
Sway closed 10: sway area	218 (189)	198 (0-799)	500 (445)	325 (8-1,988)	**0.0012**	**0.0094**
Reaction time test for non-dominant hand: average reaction time	0.198 (0.025)	0.198 (0.137-0.236)	0.218 (0.035)	0.212 (0.163-0.318)	**0.0463**	0.1234
Dominant foot: sway area	568 (300)	570 (18-1,169)	1107 (1,001)	698 (9-4,203)	**0.0394**	0.1234
Non-dominant foot: sway area	538 (452)	419 (13-1,713)	842 (672)	685 (6-3035)	**0.0287**	0.1147

*Significant values are indicated in bold*.

On the other hand, a subset of measures lost their significance upon correction for multiple testing; reaction time test for non-dominant hand (average reaction time) (*p* = 0.0463), dominant foot (sway area) (*p* = 0.0394) and non-dominant foot (sway area) (*p* = 0.0287).

### Impact of Age, CGG Repeat Number, Expression Levels of *FMR1*, *ASFMR1*, and *ASFMR1* Splice Isoform mRNAs on CATSYS Measures

**FIGURE 1:**
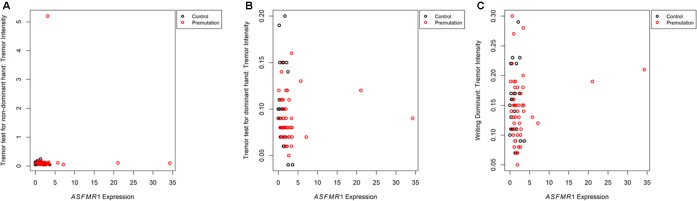
Plots showing the correlation between the expression level of the *ASFMR1* spicing isoform 131 bp *mRNA* and tremor intensity in the non-dominant hand **(A)**, tremor intensity in the dominant hand **(B)**, and tremor intensity while writing with dominant hand **(C)** in controls compared to premutations without FXTAS.

A significant correlation between the non-dominant hand tremor intensity and the *ASFMR1* 131 bp splicing isoform was observed in non-FXTAS premutation carriers (*p* = 0.0304) but not in controls (*p* = 0.3363) (**Figure 1A**). The expression level of *ASFMR1* 131 bp splicing isoform showed a significant inverse correlation with the non-dominant hand tremor intensity (-0.44) with the higher splicing isoform expression levels of this being associated with lower non-dominant hand tremor intensity score.

Correlation between *ASFMR1* 131 bp splicing and two CATSYS performance tasks, tremor intensity of dominant hand (*p* = 0.0489) (**Figure 1B**), and tremor intensity while writing with dominant hand (*p* = 0.0268) (**Figure 1C**) lost significance after correction for multiple testing.

None of the CATSYS measures showed any statistically significant association with age, CGG repeat number, expression levels of *FMR1*, or *ASFMR1* mRNAs.

### Expression Levels of Transcripts at the *FMR1* Locus

**FIGURE 2:**
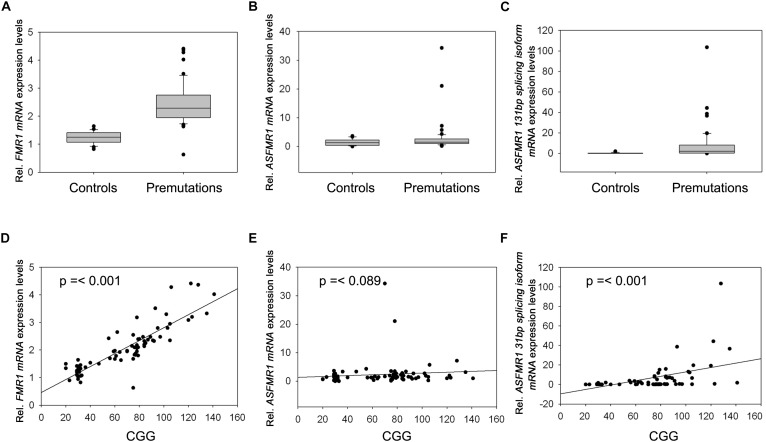
Box plots showing the expression of *FMR1* **(A)**, *ASFMR1* **(B)**, and *ASFMR1* 131 bp splicing isoform **(C)** in premutation carriers compared to controls. Correlation between CGG repeat number and the expression levels of **(D)** *FMR1*, **(E)** *ASFMR1*, and **(F)** *ASFMR1* 131 bp splicing isoform mRNAs.

Premutation carriers (with and without FXTAS) showed significantly elevated expression of both *FMR1* and *ASFMR1* 131 bp splicing isoform mRNA compared to controls with a *p* < 0.0001 in both cases (**Figures 2A,C** and **Table 4**). No significant elevation was observed for the *ASFMR1* mRNA (**Figure 2B** and **Table 4**).

**Table 4:** Summary of expression by group.

	Normal		Premutation		*p*-Value	Adjusted *p*-value
	Mean (*SD*)	Median (range)	Mean (*SD*)	Median (range)		
*FMR1*	1.24 (0.21)	1.25 (0.83–1.64)	2.44 (0.74)	2.29 (0.63–4.41)	**<0.0001**	**<0.0001**
*ASFMR1*	1.38 (1.09)	1.33 (0–3.64)	2.83 (5.35)	1.47 (0.14–34.29)	0.0889	>0.9999
*ASFMR1* 131 bp splicing isoform	0.24 (0.52)	0.02 (0–1.95)	8.14 (16.75)	2.04 (0–103.53)	**<0.0001**	**<0.0001**

*Significant values are indicated in bold*.

None of the molecular measures were significantly correlated with age. As expected, the CGG repeat number was significantly correlated with the expression of both *FMR1* (*p* < 0.0001) and *ASFMR1* 131 bp splicing isoform mRNAs (*p* = 0.001) (**Table 5** and **Figures 2D–F**) but not for the *ASFMR1* mRNA (**Figure 2E** and **Table 5**).

**Table 5:** Correlations between molecular measures and CGG repeat numbers.

	Normal			Premutation		
	Correlation	*p*-Value	Adjusted *p*-value	Correlation	*p*-Value	Adjusted *p*-value
*FMR1*	0.13	0.542	>0.9999	0.76	**<0.0001**	**<0.0001**
*ASFMR1*	0.03	0.8659	>0.9999	0.09	0.5046	0.5046
*ASFMR1* 131 bp splicing isoform	0.04	0.8424	>0.9999	0.46	**0.0006**	**0.001**

*Significant values are indicated in bold*.

**FIGURE 3:**
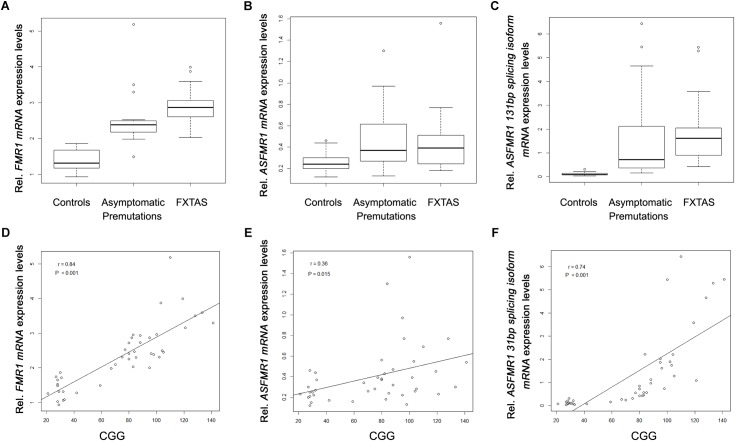
Box plots showing differential expression levels of *FMR1* **(A)**, *ASFMR1* **(B)** and *ASFMR1* 131 bp splicing isoform **(C)** mRNAs in controls compared to non-FXTAS premutation carriers and FXTAS premutation carriers. *FMR1, ASFMR1*, and *ASFMR1* 131 bp splicing isoform mRNAs expression levels, as function of the CGG repeat number, are shown in **D**, **E** and **F** respectively.

When analyzing the trios, as expected we observed a significant higher expression of *FMR1* mRNA in the premutation carriers with FXTAS and without FXTAS compared to control subjects (**Figure 3A**; *p* < 0.001 and *p* < 0.001, respectively, Sellier et al., 2014). In addition, we observed significantly higher expression level of *ASFMR1* 131 bp splicing isoform mRNA in both non-FXTAS premutation subjects [*p* = 0.003, Mean (*SD*) = 1.7 (2.1)] and subjects with FXTAS [*p* = 0.004, Mean (*SD*) = 1.9 (1.6)] compared to control subjects [Mean (*SD*) = 0.1 (0.1)] (**Figure 3C**). However, the *ASFMR1* expression levels between groups did not reach statistical significance (**Figure 3B**). These three molecular measures were significantly correlated with the number of CGG repeats (**Figures 3D–F**).

## Discussion

This study aimed to identify clinical and molecular measures that might associate with development of FXTAS in male Fragile X premutation carriers. CATSYS is among the helpful tools to distinguish between premutation carriers with and without FXTAS and controls because it allows for quantitative documentation of neuromotor and deficits with minimal operating training and time (Aguilar et al., 2008). Specifically, among the measures found useful, are those that are considered components of the key FXTAS phenotypic characteristics such as tremor intensity, postural tremors, and postural sway (Aguilar et al., 2008; Narcisa et al., 2011). Our previous reports (Aguilar et al., 2008; Narcisa et al., 2011), looking specifically at the CATSYS measure in *FMR1* premutation carriers with and without FXTAS and, unaffected controls, found the method to be sufficiently accurate for the cause. In addition, the 2008 study by Allen colleagues showed a high clinical usefulness of the battery.

In this study, we showed that three CATSYS task performance measures were significantly different in non-FXTAS male premutation carriers compared to controls. Two of them were measures of postural sway, which are associated with ataxia. Our results appear to be consistent with those of Narcisa et al. (2011), as both showed that non-FXTAS premutation carriers had higher postural sway compared to controls. However the significance of this effect was lost when correcting for multiple testing. Several factors could explain these findings including the age group of the non-FXTAS participants (mean = 59.3) compared to the mean of 52.89 in the Narcisa et al. (2011) study. Further, gender might be another contributing factor, where Narcisa et al. (2011) included females only, whereas, our study had males only. Hence, a subset of our subjects might be showing signs of ataxia because of being older males.

Importantly, this study aimed to identify molecular biomarkers at the *FMR1* locus (*FMR1*, *ASFMR1*, and *ASFMR1* 131 bp splicing isoform) that may correlate with early emergence of movement abnormalities in premutation carriers without FXTAS and might be helpful as potential biomarkers in longitudinal studies. We found an inverse correlation between both *ASFMR1* 131 bp splice isoform and tremor intensity for the non-dominant hand. Thus, using a combination of molecular and clinical phenotypic measures could help to identify changes in premutation carriers most at risk for developing FXTAS.

Interestingly, the expression of *ASFMR1* 131 bp splice isoform mRNA was higher in premutation carriers overall compared to controls, but it did not distinguish between non-FXTAS and FXTAS premutation carrier groups. The function, if any, of this splicing isoform is currently unknown and the dysregulation of the alternative splicing process and the levels of expression of the *ASFMR1* could be part of the pathogenesis of FXTAS as could be the case for the *FMR1* gene (Tseng et al., 2017). Moreover, it was previously found that RAN translation, which is one of the mechanisms proposed to explain the pathogenesis of FXTAS, also occurs in the antisense direction generating novel proteins that accumulate in ubiquitinated inclusions in FXTAS patients (Krans et al., 2016) further supporting the potential role of the antisense transcript in FXTAS. Further studies are needed to shed lights on the significance of increased expression levels of this isoforms in premutation carriers.

This study demonstrates that sensitive postural sway and tremor tests might be used in early identification of premutation carriers at risk for FXTAS, however, future longitudinal analyses with larger sample sizes are needed to confirm this hypothesis. Objective and sensitive movement measures should also be useful for monitoring disease progression, severity, and response to intervention. Further studies are warranted to further assess the correlation between the clinical and molecular measures to confirm our observations in this study.

## Author Contributions

RAO drafted the manuscript and contributed to the data analysis. FT designed the study, contributed to the data analysis, and writing the manuscript. DH and SR contributed to the design, implementation of the clinical assessments, and writing the manuscript. H-TT carried out the expression experiments and provided figures and methods for the manuscripts. AS contributed to the clinical analysis and writing the manuscript. BD-J carried out the statistical analysis and contributed to writing the manuscript.

## Conflict of Interest Statement

FT received funding from Asuragen, Inc. and Zynerba. The remaining authors declare that the research was conducted in the absence of any commercial or financial relationships that could be construed as a potential conflict of interest.
